# Health-Related Quality of Life after Hip and Knee Arthroplasty Operations

**DOI:** 10.1177/1457496920952232

**Published:** 2020-08-31

**Authors:** Hannu J. A. Miettinen, Ninna Mäkirinne-Kallio, Heikki Kröger, Simo S. A. Miettinen

**Affiliations:** Department of Orthopaedics, Traumatology and Hand Surgery, Kuopio University Hospital, Kuopio, Finland; Department of Development and Quality, Kuopio University Hospital, Kuopio, Finland; Department of Orthopaedics, Traumatology and Hand Surgery, Kuopio University Hospital, Kuopio, Finland; Faculty of Health Sciences, University of Eastern Finland, Kuopio, Finland; Department of Orthopaedics, Traumatology and Hand Surgery, Kuopio University Hospital, P.O. Box 1777, Kuopio, 70211, Finland

**Keywords:** Orthopedics, arthroplasty, outcome assessment, total knee arthroplasty, total hip arthroplasty, 15D, prospective studies, quality of life

## Abstract

**Background and objective:**

The aim of this study was to investigate the health-related quality of life before and after a hip and a knee arthroplasty operation using a 15D instrument and to compare these scores to the Finnish control population 15D scores.

**Methods:**

The pre- and post-operative data of 15D were prospective collected from the patients undergoing total hip arthroplasty or total knee arthroplasty at the Kuopio University Hospital. Post-operative data were collected at 6 and 12 months after the operation.

**Results:**

The mean change of the 15D score after hip arthroplasty was +0.062 and after knee arthroplasty, it was +0.033 at the 12-month follow-up (*p* < 0.001). Total hip arthroplasty patients of all ages reached the control population 15D scores at the 12-month follow-up. Of the total knee arthroplasty patients, only patients aged >75 years and males aged 55–64 years did reach control population 15D scores at the 12-month follow-up. Patients experienced a statistically significant improvement in mobility, vision, sleeping, usual activities, discomfort and symptoms, distress, and vitality (*p* < 0.05).

**Conclusions:**

Successful hip and knee arthroplasty operations improve patients’ health-related quality of life. According to this study, hip arthroplasty improves the health-related quality of life more than knee arthroplasty.

## Introduction

Hip and knee osteoarthritis (OA) causes pain and the restriction of the movement of the affected joint and the worsening of the quality of life^1^. The effective surgical treatment for OA is an arthroplasty^2^. Primary total hip arthroplasty (THA) and total knee arthroplasty (TKA) have been shown to decrease joint pain and improve joint movement^3^. Furthermore, arthroplasty has shown general positive changes in the quality of life, not only in the improvement of operated hip and knee joints^4–6^.

Patient-reported outcome measurements (PROMs) and quality-adjusted life years (QALYs) have started to play a more significant role in assessing the effectiveness of healthcare interventions. QALY is a health measure that takes both survival and health-related quality of life (HRQoL) of an individual into account^7,8^. The 15D instrument is a common research method to investigate changes in HRQoL^9,10^. This investigation method permits the comparison of HRQoL of the study group to standardize age and gender in the control population.

The aim of this study was to investigate the HRQoL of the patients having the THA or the TKA. This was done using the 15D instrument, and the results were compared for age and gender in the standardized Finnish control population. The control population standardized values in this study were harvested from the Health 2011 Survey in Finland^11^.

## Material and Methods

### Study Design

This was a retrospective single-center (Kuopio University Hospital (KUH)) study. The primary aim was to compare the hip and knee arthroplasty patients’ HRQoL before and after operation using the 15D PROM. The secondary aim was to compare arthroplasty patients’ 15D levels to the standardized general control population’s 15D levels^11^.

### Patients

A total of 3558 arthroplasties (1364 THAs and 2194 TKAs) were performed between June 2012 and October 2015 in the KUH. Patients were identified from our department’s longitudinal database. All patients having an arthroplasty were asked to fill in the 15D questionnaire 2 weeks prior to the arthroplasty, and the follow-up questionnaires were collected at 6 and 12 months after the arthroplasty. Patients responded to the baseline questionnaire during the pre-operative visit using a paper or an electronic response method. The follow-up questionnaires were sent to patients according to their pre-determined choice, either by e-mail or paper questionnaire. The scores of the 15D were linked to the patients’ demographic characteristics (age, gender, and date of surgery); diagnostic and operation codes were harvested from the hospital’s database by applying personal identification numbers. The control group’s 15D scores were harvested from the large Finnish population cohort study, where the participants have been selected by systematic sampling^11^. For this study, each respondent was given a population reference value corresponding to their gender and age, which formed adjusted control groups for comparison. The control group patients were randomly selected with a ratio of 1:1.

### PROM 15D

PROMs are instruments designed to measure the outcomes of interventions concerning patients’ experiences to a given treatment. PROMs can consider general life experiences or be disease-specific. PROMs allow for the comparison of various medical conditions and interventions^12^. The 15D is a generic utility-based instrument for measuring HRQoL among adults (age >16 years) and so far, it has been quoted in more than 400 international publications^13^. The 15D questionnaire is a suitable instrument for the examination of the general HRQoL of hip and knee arthroplasty patients^13^. The 15D is well known in Scandinavian, and in Finland, it is routinely used in hospitals to evaluate HRQoL of different types of patient groups, like arthroplasty patients in our hospital.

The 15D evaluates the quality of life in 15 different dimensions of life (mobility, vision, hearing, breathing, sleeping, eating, speech, excretion, usual activities, mental function, discomfort and symptoms, depression, distress, vitality, and sexual activity)^9^. Each dimension has five ordinal levels which best describes the patient’s present health status. The 15D is a profile and index measurement tool (scale 0–1), with a clinically significant value of variation at ±0.015^9^. The index value between the variation is classified into five verbal classifications as follows: much better, slightly better, much the same, slightly worse, and much worse^10^. In this study, the Finnish version of the 15D was filled manually or electrically by the patient. The Finnish set of preference weights was used to generate the 15D index on a 0–1 scale, and the algorithm provides scores ranging from 1.0 to 0.160^9^. Regression analysis has been used to estimate the minimum important change/difference for improvement/deterioration of 15D (defined as the lower/upper limit of 99.9% confidence interval (CI) of the regression coefficient, standardized for baseline HRQoL), and the generic minimum important change/difference in the 15D scores is ±0.015^10^.

### Statistical Analysis

The primary aim was to compare the data from the 15D questionnaires collected prior to the index operation (baseline) to 12 months post-operatively collected data and to control population data. The results are presented using means, percentages, standard deviations (SDs), and 95% CIs. The changes in the 15D score were estimated according to the minimal important difference ±0.015^10^. The statistical significance of the differences between the means of the continuous variables and in the dimensional responses between the measuring points was tested with a one sample or a paired sample *t*-test and an independent sample *t*-test. All *p* values ⩽0.05 were considered statistically significant. All data were analyzed using SPSS (SPSS Inc., Chicago, IL, USA; Ver 25.0.0, IBM).

### Ethical Approval

All procedures performed in studies involving human participants were in accordance with the ethical standards of the institutional and/or national research committee and with the 1964 Helsinki Declaration and its later amendments or comparable ethical standards. The Ethical Board of KUH approved the study (172/13.02.00/172/2019) and the Organizational Board of KUH gave permission for the study (19/2019).

### Informed Consent

Informed consent was obtained from all individual participants included in the study.

## Results

A total of 1819 out of a total of 3558 (51%) of all the arthroplasty patients operated on during the study period in the KUH answered the questionnaires at baseline. Of these, the questionnaires were completed at 6 months by 1324 out of 1819 (73%) patients and at 12 months by 1300 out of 1819 (71%) patients. Patients who had completed and fully filled in all the 15D dimensions were included in this study. There were a total of 802 THA and 1017 TKA patients. The mean age of the patients was 67.1 years (SD 9.9 and range 21–95). Regarding the gender of the patients, 40% were male and 60% were female.

The baseline level 15D score was statistically significantly lower in patients waiting for a THA or a TKA compared to the standardized control population groups in all age and gender subgroups (Tables 1 and 2). The change of the 15D score from the baseline level to the 6-month follow-up in the THA patients was +0.064 (*p* < 0.001) and at 12 months, it was +0.062 (*p* < 0.001) (Table 3). In the TKA patients, the change of the 15D score from the baseline level to 6 months was +0.032 and at 12 months, it was +0.033 (*p* < 0.001) (Table 3). In the subgroup analyses in terms of age and gender, male and female THA patients in all age groups reached the control population 15D scores at the 12-month follow-up (*p* > 0.05) (Table 1). In addition, male THA patients aged >75 years exceeded control population 15D scores at the 12-month follow-up (*p* = 0.016) (Table 1). Female TKA patients aged <75 years did not reach the control population 15D scores at the 12-month follow-up (*p* < 0.05), however only patients aged >75 years did (*p* = 0.079) (Table 2). Male TKA patients had a lot of variation in results as patients aged <55 years did not reach the control population 15D scores at the 12-month follow-up (*p* = 0.019), patients aged 55–64 years reached (*p* = 0.114), patients aged 65–74 years did not reach (*p* = 0.020) while patients aged >75 years reached (*p* = 0.565) (Table 2), respectively.

**Table 1.** The 15D scores of the total hip arthroplasty patients in the age and gender groups compared to the standardised control population.

	Age groups (years)											
	30–44		45–54		55–64		65–74		>75		All	
	Mean (*n*)	*p* value	Mean (*n*)	*p* value	Mean (*n*)	*p* value	Mean (*n*)	*p* value	Mean (*n*)	*p* value	Mean (*n*)	*p* value
Gender												
Male												
Baseline	0.762 (9)	<0.001	0.849 (36)	<0.001	0.830 (74)	<0.001	0.839 (88)	<0.001	0.830 (41)	0.971	0.834 (249)	<0.001
12-month F-U	0.887 (9)	0.185	0.911 (36)	0.054	0.905 (74)	0.235	0.899 (88)	0.886	0.865 (41)	0.016	0.896 (249)	0.369
Control population	0.950 (9)		0.940 (36)		0.920 (74)		0.900 (88)		0.830 (41)		0.902 (248)	
Female												
Baseline	0.806 (7)	<0.001	0.844 (27)	<0.001	0.854 (78)	<0.001	0.833 (134)	0	0.799 (74)	<0.001	0.831 (320)	<0.001
12-month F-U	0.891 (7)	0.396	0.944 (27)	0.213	0.914 (78)	0.419	0.903 (134)	0.614	0.830 (74)	0.491	0.892 (320)	0.677
Control population	0.940 (9)		0.930 (27)		0.920 (78)		0.900 (134)		0.840 (74)		0.894 (320)	

F-U: follow-up.

**Table 2.** The 15D scores of the total knee arthroplasty patients in the age and gender groups compared to the standardised control population.

	Age groups (years)											
	30–44		45–54		55–64		65–74		>75		All	
	Mean (*n*)	*p* value	Mean (*n*)	*p* value	Mean (*n*)	*p* value	Mean (*n*)	*p* value	Mean (*n*)	*p* value	Mean (*n*)	*p* value
Gender												
Male												
Baseline	0.842 (1)	n/a	0.851 (22)	<0.001	0.858 (87)	<0.001	0.851 (117)	<0.001	0.839 (53)	0.456	0.851 (280)	<0.001
12-month F-U	0.806 (1)	n/a	0.890 (22)	0.019	0.904 (87)	0.114	0.877 (117)	0.020	0.839 (53)	0.565	0.879 (280)	0.005
Control population	0.950 (1)		0.940 (22)		0.920 (87)		0.900 (117)		0.830 (53)		0.896 (280)	
Female												
Baseline	0.815 (1)	n/a	0.842 (36)	<0.001	0.844 (130)	<0.001	0.847 (158)	<0.001	0.836 (126)	0.609	0.842 (451)	<0.001
12-month F-U	1.00 (1)	n/a	0.889 (36)	0.013	0.894 (130)	<0.001	0.881 (158)	0.017	0.855 (126)	0.079	0.878 (451)	0.003
Control population	0.940 (1)		0.930 (36)		0.920 (130)		0.900 (158)		0.840 (126)		0.892 (451)	

F-U: follow-up; n/a: not applicable.

**Table 3.** The 15D scores collected before the operation, 6 and 12 months post-operatively.

	15D					
	Baseline level	6-month F-U	*p* value	12-month F-U	*p* value	Control population
THA						
*n*	802	574		569		
Mean 15D (SD, 95% CI)	0.824	0.888		0.886		0.901
Change of the mean 15D (SD, 95% CI)		0.064 (0.076, 0.058–0.070)	<0.001	0.062 (0.083, 0.055–0.069)	<0.001	
TKA						
*n*	1017	750		731		
Mean 15D (SD, 95% CI)	0.837	0.869		0.870		0.896
Change of the mean 15D (SD, 95% CI)		0.032 (0.074, 0.026–0.037)	<0.001	0.033 (0.076, 0.028–0.039)	<0.001	

F-U: follow-up; THA: total hip arthroplasty; SD: standard deviation; CI: confidence interval; TKA: total knee arthroplasty.

**Fig. 1.**
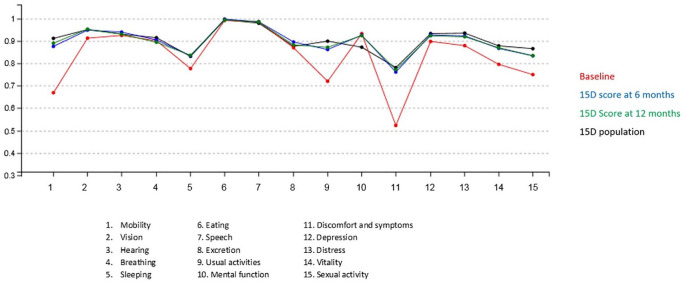
The 15D levels (scale 0–1 and 15 dimensions) of the total hip arthroplasty (THA) patients.

**Fig. 2.**
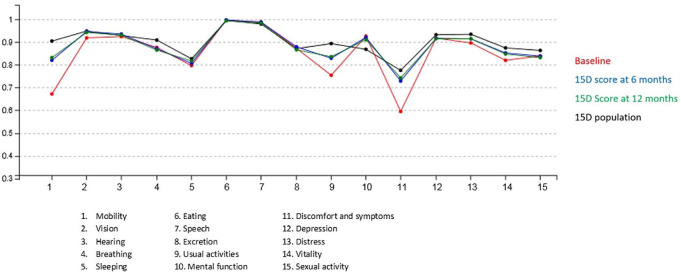
The 15D levels (scale 0–1 and 15 dimensions) of the total knee arthroplasty (TKA) patients.

There were statistically significantly improvements of the 15D scores in mobility, vision, sleeping, usual activities, symptoms, depression, distress, vitality, and sexual life in the THA patient group (Table 4 and Fig. 1). In the TKA patient group, there were statistically significantly improvements of the 15D scores in mobility, vision, sleeping, usual activities, discomfort and symptoms, distress, and vitality (Table 4 and Fig. 2). Interestingly, in the TKA patient group, the mental function score was statistically significantly lowered (Table 4 and Fig. 2).

**Table 4.** The 15D dimensions before the operation, at the 12-month follow-up and the change of the mean in the 0-12 follow-up time of the hip and knee arthroplasties.

	THA (*n* = 569)			*p* value	TKA (*n* = 731)			*p* value
	Baseline	12-month F-U	Change of mean in 0–12 months		Baseline	12-month F-U	Change of mean in 0–12 months	
Dimension								
Mobility	0.669	0.882	0.213	<0.001	0.672	0.835	0.163	<0.001
Vision	0.913	0.946	0.033	<0.001	0.917	0.939	0.022	<0.001
Hearing	0.921	0.929	0.008	0.123	0.919	0.925	0.006	0.214
Breathing	0.896	0.887	−0.007	0.363	0.868	0.862	−0.006	0.386
Sleeping	0.773	0.833	0.060	<0.001	0.799	0.814	0.015	0.024
Eating	0.990	0.993	0.003	0.365	0.993	0.992	−0.002	0.481
Speech	0.986	0.986	−0.002	0.549	0.986	0.981	−0.005	0.079
Excretion	0.868	0.877	0.009	0.259	0.872	0.866	−0.006	0.391
Usual activities	0.713	0.859	0.146	<0.001	0.751	0.832	0.080	<0.001
Mental function	0.927	0.918	−0.008	0.188	0.922	0.905	−0.017	0.003
Discomfort/symptoms	0.520	0.761	0.241	<0.001	0.591	0.741	0.151	<0.001
Depression	0.895	0.921	0.025	<0.001	0.916	0.915	−0.001	0.771
Distress	0.877	0.914	0.037	<0.001	0.894	0.914	0.020	<0.001
Vitality	0.791	0.864	0.073	<0.001	0.815	0.848	0.033	<0.001
Sexual activity	0.742	0.832	0.090	<0.001	0.836	0.828	−0.008	0.325

THA: total hip arthroplasty; TKA: total knee arthroplasty; F-U: follow-up.

A total of 75% of the THA patients answered that their HRQoL was much better or slightly better than before the surgery. However, 15% of the THA patients answered that their HRQoL was slightly worse or much worse. In TKA patients, a total of 64% answered that their HRQoL was much better or slightly better than before surgery and 23% answered that their HRQoL was slightly worse or much worse, respectively.

## Discussion

In this study, we found that the 15D scores of the THA patients improved in all age and gender groups and reached the control population level at the 12-month follow-up while the TKA patients’ 15D scores reached the control population level only in males aged 55–64 years and in both genders aged >75 years, respectively. Thus, we found that the THA patients had better overall results compared to the TKA patients. The findings of our study support previous studies which have shown that successful hip and knee arthroplasty operations improve the HRQoL^2–4,6,14^.

The number of THAs and TKAs will increase in the next few decades and its effectiveness should be monitored^14,15^. The most important criteria of effectiveness are the patient’s own assessment of symptomatic, functional ability, and quality of life before and after treatment. Measuring the benefits of arthroplasty for patients should be an important part of the continuous assessment of effectiveness of healthcare and quality development work. There is a myriad of PROMs available for arthroplasty patients, like EQ5D and SF36, and it is difficult to choose and implement the best PROM^16,17^. One valid tool for measuring the general quality of life after arthroplasty is the 15D^4–6,13^.

We found that patients waiting for an arthroplasty had a poorer HRQoL compared to the standardized control population. Similar results have been shown in a previous Finnish study and also in Australian and Norwegian populations^5,18,19^. Patients after a THA have shown larger improvements in pain and function, and the patients were more satisfied with the outcome of the operation than patients after a TKA^20^. Our results are similar as the THA patients had better 15D scores than the TKA patients at the 12-month follow-up. Moreover, the TKA patients’ 15D scores did not improve as much as the THA patients’ 15D scores at the 12-month follow-up which suggests that the TKA patients benefited less from the arthroplasty. Our findings are similar to previous studies which have shown that arthroplasty patients have lower HRQoL scores compared to non-arthroplasty patients of the standardized age and gender population before arthroplasty^4,5,20^. Previously, it has been shown that an increased age predicts a worse recovery of THA patients compared to younger patients^4,21^. Our study findings support these findings as the 15D scores improved more in the THA patients who were ⩾65 years aged compared to younger <65 years aged patients. We did not study THA fixation type (cemented or cementless) effect on HRQoL but in previous study, it has been shown that cementless endoprostheses achieved better short-term outcomes^22^.

Female THA patients ⩾65 years had the poorest baseline scores and their 15D scores did not reach other THA patient groups’ scores during the 12-month follow-up period. Similar results have been reported by Ackerman et al.^18^, where advanced age and being female predicted poorer results for the arthroplasty. In the TKA patients, the baseline 15D scores were similar in all the age groups. In general, the male and female TKA patients’ baseline 15D scores were higher than the THA patients’ baseline scores were, but at the 12-month follow-up, TKA patients’ scores were lower than THA patients. Moreover, in a previous study by Kauppila et al.^23^, it was shown that the TKA patients’ pre-operative baseline 15D score was strongly associated with the achieved level of HRQoL, and this presents the multifactorial nature of the health status of TKA patients.

Interestingly, the sight ability and energy level experienced were improved after arthroplasty. Probably, improved mobility without pain might improve the general well-being and this could explain these surprising findings. Also, some of these research findings may be explained by the general nature of the 15D instrument. However, more research on this topic is warranted. It is also important to use both disease-specific PROMs and general HRQoL instruments in analyzing the effectiveness of arthroplasty.

We found that 75% of the THA patients felt that their HRQoL was better after the arthroplasty but 25% of the patients did not have an improvement in their HRQoL at the 12-month follow-up. Respectively, a better HRQoL was achieved in only 64% of the TKA patients and 36% did not have an improvement in their HRQoL. A consistently worse HRQoL has been observed in patients waiting for major joint replacement compared with the population controls as well as similar results of satisfaction of the arthroplasty have been reported previously^5,17,24,25^. It is possible that patients with more severe OA symptoms did not respond to the questionnaires^24,25^. Judge et al.^24^ noticed that these non-completers had higher pain and function scores pre-operatively. This response bias could underestimate the proportion of patients responding good to the arthroplasty. More research is needed to find out why some of the patients do not benefit from the arthroplasty. In addition, in the future, it could be possible to evaluate pre-operatively, with the 15D score, those OA patients who will probably benefit from the arthroplasty and those who will not. Moreover, in the future, these kinds of general HRQoL PROMs like 15D might become an important decision factor for the surgical indication. However, in addition to PROMs, the patient’s expectations must be clearly evaluated beforehand and compared to the typical outcomes after arthroplasty. Our study did have some other limitations, since there are likely confounding variables (e.g. other diseases affecting general life experiences, somatization influence, and depression) affecting general well-being and were not included^26^. One weakness of the 15D questionnaire is the lack of a social support evaluation which has been shown to be an important part of recovery after an arthroplasty^20^. Lack of multiple PROMs designed specifically for OA and arthroplasty patients and multiple testing were not available in this study which may affect to result interpretation. Due to missing data, exclusion of some patients was made, and this could cause some bias in the results.

## Conclusion

The HRQoL of the THA patients improved in all age and gender groups and reached the control population level while the TKA patients’ HRQoL did reach the control population level only in males aged 55–64 years and in both genders aged >75 years. The THA patients had better overall results compared to the TKA patients.
